# Emergencies and How to Treat Them

**Published:** 1872-01

**Authors:** 


*Emergencies and How to Treat Them.* By Joseph N. Howe, M. D. New York; D. Appleton & Co., 1871.

The "emergencies" treated by this author are the following: hemorrhage, wounds of important organs, wounds of arteries and veins, poisoned wounds, extraction of foreign bodies, burns, scalds and effects of cold, strangulated hernia, loss of conciousness, asphyxia, sun stroke, dyspnœa œdema glottidis, convulsions, suspended animation, complications of labor, poisons.

There are chapters upon all the above "emergencies," and very well written and correct views are expressed upon the various topics. It is offered to students and practitioners, and we believe the author has combined the most recent views with the most attractive style of presenting them.

